# Blood and urine biomarkers for the diagnosis of early stages of knee osteoarthritis: A systematic review

**DOI:** 10.1002/jeo2.12105

**Published:** 2024-07-29

**Authors:** Marko Ostojic, Joao Pedro Oliveira, David Kordic, Caroline Mouton, Robert Prill, Roland Becker

**Affiliations:** ^1^ Department of Orthopaedics and Traumatology University Hospital Mostar Mostar Bosnia and Herzegovina; ^2^ Osteon Orthopedics Trauma and Sports Medicine Clinic Mostar Bosnia and Herzegovina; ^3^ Faculty of Medicine University of Coimbra Coimbra Portugal; ^4^ Orthopaedic Department, Hospitais da Universidade de Coimbra Unidade Local de Saúde de Coimbra Coimbra Portugal; ^5^ Department of Orthopaedic Surgery Centre Hospitalier Luxembourg—Clinique d'Eich Luxembourg City Luxembourg; ^6^ Center of Orthopaedics and Traumatology University Hospital Brandenburg/Havel, Brandenburg Medical School Brandenburg an der Havel Germany; ^7^ Brandenburg Medical School, Faculty of Health Sciences Brandenburg Brandenburg an der Havel Germany

**Keywords:** biomarkers, diagnose, early, knee, osteoarthritis

## Abstract

**Purpose:**

To identify biomarkers in human blood or urine at an early stage of knee osteoarthritis (OA) and to elucidate if any can accurately differentiate between healthy controls and early knee OA patients and be considered as a candidate for widespread clinical use for early diagnosis of the disease.

**Methods:**

Medline, Embase and Web of Science were screened to identify comparative studies measuring differences in blood or urine biomarkers between healthy controls and knee OA patients at an early stage (grade 1 or 2 Kellgren–Laurence). Two independent reviewers screened the abstracts for eligibility, reviewed the full texts, assessed the methodological quality and extracted the data. The Joanna Briggs Institute critical appraisal tool for diagnostic test accuracy studies was used to assess the quality of the included studies. Due to relevant heterogeneity, meta‐analysis was not appropriate.

**Results:**

Five studies met the eligibility criteria. The examined biomarkers were adropin, collagen type II metabolite, C‐terminal cross‐linked telopeptide of type II collagen, C‐terminal cross‐linked telopeptide of type I collagen, cartilage oligomeric matrix protein, matrix metalloproteinase 3, N‐terminal propeptide of procollagen type IIA, type I procollagen N‐terminal propeptides, N‐terminal osteocalcin, angiopoietin‐2, follistatin, granulocyte colony‐stimulating factor, hepatocyte growth factor, interleukin‐8, leptin, platelet‐derived growth factor‐BB, platelet endothelial cell adhesion molecule‐1, vascular endothelial growth factor and calprotectin and totalling 19 biomarkers. All of the biomarkers were studied only once in the selected papers.

**Conclusions:**

There is no reliable biomarker available to differentiate between early knee OA in patients and healthy controls, but a potential role of a cluster of biomarkers to close this gap. There are several limitations, including inappropriate study designs, small sample sizes, nonconsecutive patient groups and inadequate statistical methods for evaluating biomarker performance in studies included.

**Level of Evidence:**

Level III.

**Abbreviations**
C2C: collagen type II metabolite
COMP: cartilage oligomeric matrix protein
CTX‐I: C‐terminal cross‐linked telopeptide of type I collagen
CTX‐II: C‐terminal cross‐linked telopeptide of type II collagen
G‐CSF: granulocyte colony‐stimulating factor
HGF: hepatocyte growth factor
IL‐8: interleukin‐8
MMP‐3: matrix metalloproteinase 3
MRI: magnet resonance imaging
OA: osteoarthritis
OC: osteocalcin
PDGF‐BB: platelet‐derived growth factor‐BB
PECAM‐1: platelet endothelial cell adhesion molecule‐1
PIIANP: N‐terminal propeptide of procollagen type IIA
PINP: procollagen N‐terminal propeptides
PRISMA: Preferred Reporting Items for Systematic Reviews and Meta‐analyses
VEGF: vascular endothelial growth factor

## INTRODUCTION

Knee osteoarthritis (OA) is a progredient and disabling condition that highly impacts quality of life and is nowadays a growing socioeconomic burden [19]. Ideally, OA should be diagnosed early, before the disease irreversibly damages joint structure and function. However, OA is typically diagnosed late when symptoms are obvious and damage is irreversible.

The standard for diagnosing knee OA is the clinical examination, with standardised criteria according to the American Rheumatism Association [1]. It is generally completed with radiographs, which remain the standard to distinguish between the different stages of OA and diagnosing early OA. Joint deterioration is most commonly assessed using the Kellgren–Lawrence (KL) classification [11]. While magnetic resonance imaging (MRI) can detect early abnormalities and articular damage [18], it is expensive, time‐consuming and limited in availability.

In order to better diagnose OA at its earlier stages, over the last decades, researchers have tried to identify other precursor signs of OA, such as molecular biomarkers. By definition, the biomarker is a ‘characteristic that is objectively measured and evaluated as an indicator of normal biologic processes, pathogenic processes or pharmacologic responses to a therapeutic intervention’ [5]. With these characteristics, biomarkers could indicate the joint remodelling process occurring in OA [13]. The best candidates for the evaluation of OA are structural molecules or fragments linked to the cartilage, bone, synovium and associated metabolisms [2, 16]. However, no data confirms their accuracy in distinguishing diseased from healthy individuals, limiting their clinical use. Efficient biomarkers should be sensitive, specific, easy to obtain and inexpensive [2]. As synovial fluid biomarkers are difficult to access and require invasive methods, blood or urine samples are preferred to identify early signs of OA.

This systematic review aims to identify biomarkers in human blood or urine that can distinguish between healthy individuals and early‐stage knee OA patients. The goal is to find biomarkers that can accurately differentiate these groups and be used for widespread early diagnosis of OA.

## METHODS

**Figure 1:**
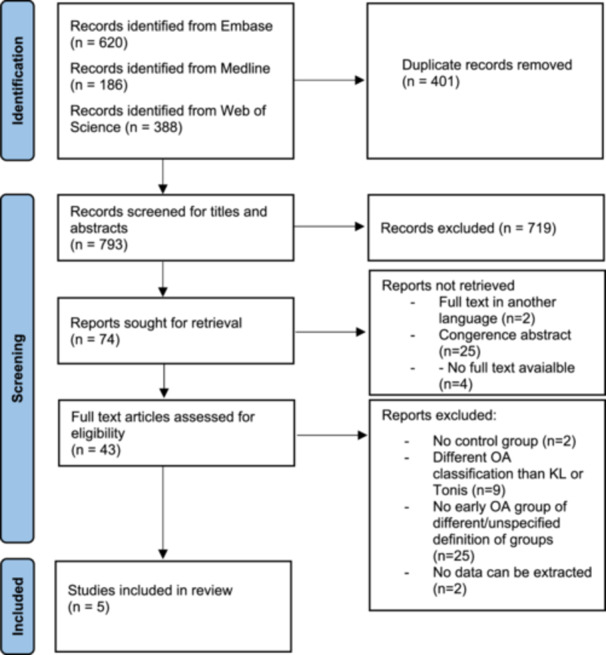
Flow chart of the study selection process. OA, osteoarthritis.

This systematic review was conducted in accordance with the author's guidelines for conducting systematic reviews and meta‐analyses [25]. Results are reported in adherence with the Preferred Reporting Items for Systematic Reviews and Meta‐analyses (PRISMA) guidelines [24]. A detailed PRISMA flow chart is provided in Figure 1. The review was not registered, nor was the protocol published.

Three medical bibliographic databases were searched for relevant publications—Medline, Embase and Web of Science. The search, which was conducted on 30 October 2022, covered titles, abstracts and keywords, and it covered all of the articles indexed in mentioned databases up to date. No filters and limits were used. The query used for Medline, Embase and Web of Science searches was as follows: (knee) AND (osteoarthritis OR arthrosis) AND (biomarker OR marker OR biomarkers OR markers) AND (blood OR serum OR plasma OR urine OR urinary) AND (early).

All records were imported and organised in the Rayyan programme [23]. Duplicates were removed, and the titles and abstracts were screened for eligibility by two independent reviewers and selected for full‐text reading. Abstracts which met the following criteria were included for full‐text review: (1) studies on human subjects, (2) studies that evaluated biomarkers in blood and/or urine, (3) studies with healthy control subjects compared with subjects with early knee OA, (4) studies that used the KL classification of knee OA (defining early OA as grade 1 and 2 and control group with a grade 0) and (5) studies in English. Disagreements between reviewers were resolved by consensus.

The selected publications with full‐text availability were imported into the Joanna Briggs Institute (JBI) Sumari programme [20] and independently reviewed by two authors to verify the inclusion criteria. Reasons for excluding papers were recorded. Papers selected for the final analysis were assessed for potential Risk of Bias by the same authors using the JBI critical appraisal tool for diagnostic test accuracy studies [26]. This tool consists of ten questions concerning internal validity for this study type and can be rated with ‘yes’, ‘no’, ‘unclear’ or ‘not applicable’ for the presence of a methodological important study characteristic. The findings of this appraisal are presented both narratively and in tabular form. We consider Scores of 9–10 as a low risk of bias, 6–8 as moderate and below 6 as a high risk of bias in case all items are applicable according to the JBI appraisal score. Missing information increases the risk of bias for the specific domain and the whole study [35]. Despite variations in methodological quality, all studies undergo data extraction and synthesis where feasible. Sensitivity analyses for exploring the impact of methodological quality on outcomes by aggregating studies with similar appraisal scores were not feasible due to high content heterogeneity and inadmissibility of data synthesis.

In both steps, in case of conflicts, a third author reviewed the publication and, after consultations with reviewers, made the final decision.

Data extraction was performed by one author into a Microsoft Excel spreadsheet. The following information was extracted: publication year, authors, country, timeframe for data collection, joint, fluid specimen (serum, plasma or urine), sample sizes, sex, age, body mass index, OA grade with KL or Tönnis classification (only for patients with grades ≤2), biomarkers investigated, results and conclusions.

## RESULTS

The initial search produced 1194 results (620 via Embase, 186 via Medline and 388 via Web of Science). Four hundred and one duplicate results were removed, and the remaining 793 articles were screened for titles and abstracts. Out of these, 74 were selected for full‐text review. In total, five articles met the criteria for qualitative synthesis (Figure 1, Table 1). All of the selected studies included both men and women. There were 19 different blood (*n* = 17) or urine (*n* = 2) biomarkers investigated. Each of them was only investigated once, preventing the authors from performing a synthesis of the data in a meta‐analysis. As classified by the ‘European Society for Clinical and Economic Aspects of Osteoporosis and Osteoarthritis’ (ESCEO) [16], biomarkers included in this review can be related to the following: collagen metabolism (collagen type II metabolite [C2C], C‐terminal cross‐linked telopeptide of type II collagen [CTX‐II], C‐terminal cross‐linked telopeptide of type I collagen [CTX‐I], N‐terminal propeptide of procollagen type IIA [PIIANP], type I procollagen N‐terminal propeptides [PINP]), other noncollagenous proteins (cartilage oligomeric matrix protein [COMP], N‐terminal osteocalcin [OC]) and other processes—mostly inflammatory (matrix metalloproteinase 3 [MMP‐3], angiopoietin‐2, follistatin, granulocyte colony‐stimulating factor [G‐CSF], hepatocyte growth factor [HGF], interleukin‐8 [IL‐8], leptin, platelet‐derived growth factor‐BB [PDGF‐BB], platelet endothelial cell adhesion molecule‐1, vascular endothelial growth factor, calprotectin). This classification was developed by a group of experts from ESCEO [16].

**Table 1:** Overview of studies included in the qualitative analysis.

Authors	Year	Country	Data collection timeframe	Source	Biomarkers	Group	Sample size	Gender (male/female)	Age	BMI (kg/m^2^)	KL grade
Gundogdu et al.	2018	Turkey	July 2017 to May 2017	Serum	Adropin	Healthy controls	30	9/21	62 ± 9	32.8 ± 6.3	0
						OA	8	Unreported	Unreported	Unreported	1
							12				2
He et al.	2014	China	March 2013 to Jun 2013	Urine	C2C	Healthy controls	20	8/12	56 ± 10	Unreported	0
						OA	31	11/20	58 ± 8	Unreported	1
							14	5/9	57 ± 9		2
Hu et al.	2022	China	2017	Urine	CTX‐II	Healthy controls	152	44/108	Males 46 ± 3; females 46 ± 4	Males 24.9 ± 3.0; females 24.2 ± 3.0	0
				Serum	COMP, MMP‐3, PIIANP, PINP, OC, CTX‐I	OA	189	43/146	Males 46 ± 3; females 47 ± 3	Males 25.4 ± 2.8; females 25.1 ± 3.0	1
Mabey et al.	2014	Thailand	Unknown	Plasma	Angiopoietin‐2, follistatin, G‐CSF, HGF, IL‐8, leptin, PDGF‐BB, PECAM‐1, VEGF	Healthy controls	15	7/8	62 ± 5	Unreported	0
						OA	Unreported	Unreported	Unreported	Unreported	2
Safa et al.	2021	Iran	Unknown	Serum	Calprotectin	Healthy controls	52	15/37	51 ± 10	26.6 ± 4.4	0
						OA	32	Unreported	Unreported	Unreported	1
							28	Unreported	Unreported	Unreported	2

*Note*: Study, patient and biomarkers characteristics.

Abbreviations: BMI, body mass index; C2C, collagen type II metabolite; COMP, cartilage oligomeric matrix protein; CTX‐I, C‐terminal cross‐linked telopeptide of type I collagen; CTX‐II, C‐terminal cross‐linked telopeptide of type II collagen; G‐CSF, granulocyte colony‐stimulating factor; HGF, hepatocyte growth factor; IL‐8, interleukin‐8; KL, Kellgren–Lawrence; MMP‐3, matrix metalloproteinase 3; OC, N‐terminal osteocalcin; PDGF‐BB, platelet‐derived growth factor‐BB; PECAM‐1, platelet endothelial cell adhesion molecule‐1; PIIANP, N‐terminal propeptide of procollagen type IIA; PINP, type I procollagen N‐terminal propeptides; VEGF, vascular endothelial growth factor.

The JBI critical appraisal scores ranged from 5 to 7, averaging 6.2 (Table 2). There is a moderate to high risk of bias for most selected studies. However, they presented with a systematic bias in the patient selection process as it was unsure whether the patients were consecutive or part of a random sample. Four out of five studies were based on a case–control design. Finally, the interval between tests (radiographs to determine KL grade and blood/urine sample) was unsure for four out of five studies.

**Table 2:** Critical appraisal of the five studies included at the final stages using the JBI critical appraisal tool for diagnostic test accuracy studies.

	Q1	Q2	Q3	Q4	Q5	Q6	Q7	Q8	Q9	Q10	Score
Gundogdu et al.	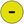	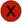	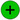	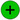	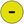	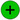	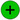	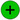	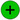	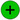	7
He et al.	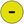	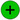	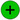	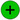	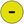	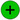	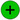	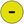	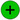	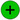	7
Hu et al.	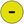	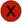	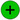	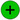	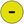	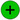	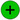	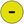	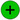	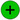	6
Mabey et al.	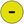	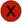	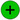	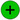	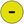	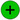	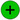	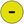	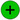	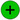	6
Safa et al.	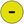	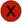	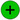	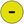	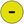	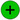	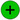	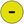	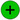	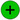	5

*Note*: (Q1) Was a consecutive or random sample of patients enroled? (Q2) Was a case–control design avoided? (Q3) Did the study avoid inappropriate exclusions? (Q4) Were the index test results interpreted without knowledge of the results of the reference standard? (Q5) If a threshold was used, was it prespecified? (Q6) Is the reference standard likely to correctly classify the target condition? (Q7) Were the reference standard results interpreted without knowledge of the results of the index test? (Q8) Was there an appropriate interval between the index test and the reference standard? (Q9) Did all patients receive the same reference standard? (Q10) Were all patients included in the analysis?

Abbreviations: 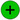, yes, 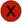, no, 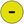, unclear or not available; JBI, Joanna Briggs Institute.

Due to the low number of studies included and the fact that each biomarker was only reported once, a meta‐analysis could not be conducted. Specific results for each study are thus presented separately in Table 3.

**Table 3:** Overview of studies included in the qualitative analysis.

Authors	Source	Biomarkers	Unit	Group	Level	Conclusions	Other findings related to OA
Gundogdu et al.	Serum	Adropin	ng/mL	Healthy controls	1.102 ± 0.106	Serum adropin level: significantly ↘ in KL grades 1 and 2 compared with healthy controls	Strong negative correlation between serum adropin and TNF‐α (pro‐inflammatory cytokines)
				OA grade 1	1.020 ± 0.031		
				OA grade 2	0.939 ± 0.043		
He et al.	Urine	C2C	pg/mL	Healthy controls	218.341 ± 22.270	Urine C2C level: KL grade II > KL grade I group or control group/concentrations of C2C in KL grade I not significantly different from the control group	Important role of trace elements, Fe, Cu and Zn in the progression of KOA
				OA grade 1	219.098 ± 21.986		
				OA grade 2	257.319 ± 24.056		
Hu et al.	Urine	CTX‐II	ng/mL	Healthy controls	♂ 12.60 ± 4.92 ♀ 10.26 ± 4.51	♂ Serum COMP (*p* < 0.001) higher in OA patients than in controls ♀ Serum COMP (*p* < 0.001), PINP (*p* < 0.001), OC (*p* = 0.005) and urine CTX‐II (*p* < 0.001) higher in OA patients than in controls. Female patients had lower serum PINP (*p* < 0.001), CTX‐I (*p* < 0.001), OC (*p* = 0.001) compared to male patients	Sex differences exist in KOA
				OA grade 1	♂ 16.00 ± 5.02 ♀ 15.50 ± 5.94		
	Serum	COMP	ng/mL	Healthy controls	♂ 152.87 ± 76.58 ♀ 144.33 ± 72.43		
				OA grade 1	♂ 264.40 ± 125.41 ♀ 272.00 ± 144.91		
		MMP‐3	ng/mL	Healthy controls	♂ 7.43 ± 2.97 ♀ 7.04 ± 2.49		
				OA grade 1	♂ 7.07 ± 2.21 ♀ 7.66 ± 3.13		
		PIIANP	ng/mL	Healthy controls	♂ 8.41 ± 2.43 ♀ 8.47 ± 2.08		
				OA grade 1	♂ 8.66 ± 1.73 ♀ 8.43 ± 2.61		
		PINP	ng/mL	Healthy controls	♂ 47.57 ± 14.63 ♀ 41.44 ± 13.25		
				OA grade 1	♂ 50.02 ± 22.38 ♀ 35.71 ± 11.53		
Hu et al.	Serum	OC	ng/mL	Healthy controls	♂ 16.62 ± 4.45 ♀ 15.40 ± 4.99	♂ Serum COMP (*p* < 0.001) higher in OA patients than in controls ♀ Serum COMP (*p* < 0.001), PINP (*p* < 0.001), OC (*p* = 0.005) and urine CTX‐II (*p* < 0.001) higher in OA patients than in controls. Female patients had lower serum PINP (*p* < 0.001), CTX‐I (*p* < 0.001), OC (*p* = 0.001) compared to male patients	Sex differences exist in KOA
				OA grade 1	♂ 18.51 ± 8.40 ♀ 16.62 ± 4.45		
		CTX‐I	pg/mL	Healthy controls	♂ 488.71 ± 165.55 ♀ 340.58 ± 163.49		
				OA grade 1	♂ 549.27 ± 360.50 ♀ 320.34 ± 190.88		
Mabey et al.	Plasma	Angiopoietin‐2	pg/mL	Healthy controls	173.7 (132.4–198.3)	Results are expressed in median (Q1–Q3). Median plasma concentrations of G‐CSF, HGF, angiopoietin‐2, follistatin and IL‐8 were significantly higher in early OA patients than in controls (*p* < 0.001). Plasma PECAM‐1 (*p* = 0.01) and VEGF levels (*p* = 0.002) were also significantly greater in early OA compared with controls	Plasma levels of follistatin further decreased in advanced KOA, and plasma levels of angiopoietin‐2 further increased in advanced KOA patients.
				OA grade 2	294.1 (237.0–307.4)		
		Follistatin	pg/mL	Healthy controls	80.5 (67.4–94.8)		
				OA grade 2	173.9 (131.8–204.2)		
		G‐CSF	pg/mL	Healthy controls	48.4 (32.3–62.4)		
				OA grade 2	90.2 (83.5–129.8)		
		HGF	pg/mL	Healthy controls	69.0 (46.8–100.4)		
				OA grade 2	251.0 (137.3–295.1)		
		IL‐8	pg/mL	Healthy controls	3.8 (2.1–4.8)		
				OA grade 2	9.8 (7.7–11.4)		
		Leptin	pg/mL	Healthy controls	1185.1 (88.8–1265.1)		
				OA grade 2	1316.2 (778.4–2068.4)		
		PDGF‐BB	pg/mL	Healthy controls	19.9 (19.9–19.9)		
				OA grade 2	30.2 (5.7–31.3)		
Mabey et al.	Plasma	PECAM‐1	pg/mL	Healthy controls	159.7 (116.0–168.8)	Results are expressed in median (Q1–Q3). Median plasma concentrations of G‐CSF, HGF, angiopoietin‐2, follistatin and IL‐8 were significantly higher in early OA patients than in controls (*p* < 0.001). Plasma PECAM‐1 (*p* = 0.01) and VEGF levels (*p* = 0.002) were also significantly greater in early OA compared with controls	Plasma levels of follistatin further decreased in advanced KOA, and plasma levels of angiopoietin‐2 further increased in advanced KOA patients.
				OA grade 2	488.7 (387.5–540.5)		
		VEGF	pg/mL	Healthy controls	7.6 (5.8–9.6)		
				OA grade 2	15.6 (10.9–18.9)		
Safa et al.	Serum	Calprotectin	ng/mL	Healthy controls OA grade 1 OA grade 2	901 ± 875 3740 ± 2728 3100 ± 2084	Serum calprotectin levels were significantly higher in KOA patients than in healthy control subjects	Not applicable

*Note*: Study, patient and biomarkers characteristics.

Abbreviations: C2C, collagen type II metabolite; COMP, cartilage oligomeric matrix protein; CTX‐I, C‐terminal cross‐linked telopeptide of type I collagen; CTX‐II, C‐terminal cross‐linked telopeptide of type II collagen; G‐CSF, granulocyte colony‐stimulating factor; HGF, hepatocyte growth factor; IL‐8, interleukin‐8; KL, Kellgren–Lawrence; KOA, knee OA; MMP‐3, matrix metalloproteinase 3; OA, osteoarthritis; OC, N‐terminal osteocalcin; PDGF‐BB, platelet‐derived growth factor‐BB; PECAM‐1, platelet endothelial cell adhesion molecule‐1; PIIANP, N‐terminal propeptide of procollagen type IIA; PINP, type i procollagen N‐terminal propeptides; TNF‐α, tumour necrosis factor‐α; VEGF, vascular endothelial growth factor.

## DISCUSSION

Although this systematic review highlighted several potential biomarkers to detect early knee OA from blood or urine samples, no diagnostic biomarker could be confirmed as being accurate to differentiate between healthy controls and patients with knee OA at its early stage. Included studies indeed were inappropriately designed to serve this purpose, including small sample sizes, nonconsecutive groups of patients and inappropriate statistical methods to quantify the overall ability of each biomarker to classify diseased and nondiseased individuals correctly. Despite the strong support of the scientific literature for the necessity to use biomarkers for understanding OA, there is still a lack of adequate studies using biomarkers as a diagnostic tool for knee OA at their early stages.

Most of the biomarkers investigated were from cartilage catabolism, which is considered to be the main driving process of OA. Previous systematic reviews also concluded that matrix degradation biomarkers have greater diagnostic applicability in comparison with other biochemical marker categories, such as matrix synthesis or synovium and bone metabolism [16, 27, 34]. In general, type II collagen metabolism biomarkers are associated with the metabolism of cartilage, while type I collagen is associated with subchondral bone metabolism [7]. Aggrecan metabolism biomarkers are associated with cartilage turnover as well [16]. Some of the biomarkers were inflammatory biomarkers, which are generally nonspecific for OA. Also, there were some other nonspecific biomarkers, like leptin, which is adipose‐derived or adropin, a lipid and glucose metabolism regulator protein.

A study included in this systematic review of final data extraction examined biomarkers of bone (PINP, OC and CTX‐I) and cartilage turnover (PIIANP, COMP, CTX‐II and MMP‐3) [10]. The results found that serum COMP and urine CTX‐II showed positive associations with premenopausal early knee OA, while serum PINP had a negative association. However, in male patients, only serum COMP was higher than control. Gender differences were found, which is an important finding of the aforementioned study. COMP and CTX‐II were, by some previous systematic reviews, considered the most promising biomarkers for knee OA [16, 34].

COMP is a noncollagenous extracellular matrix glycoprotein that is produced by articular chondrocytes. It is believed to be a reliable biomarker of cartilage turnover, and it can be sensitive to asymptomatic early knee OA [27]. Some authors conclude that serum COMP might not be a valuable indicator because other cartilaginous tissues also produce small amounts of COMP in their cartilage turnover. COMP is also present in rheumatoid arthritis, which makes it less specific for OA [33]. However, recent studies have concluded that COMP is associated with OA diagnosis but also with the weight of the subjects, which makes it less specific, with obesity being one of the contributors to OA progression [32]. COMP is still not used in clinical practice [28].

CTX‐II is MMP generated, and it is localised to the bone–cartilage interface and damaged articular cartilage of the OA knee in humans. It is observed that CTX‐II is associated with knee OA progression and the likelihood of total joint replacement [13]. Interestingly, CTX‐II is one of the biomarkers at the end of the pathways of tissue destruction, which may be more specific for a particular tissue, articular cartilage, in this case. Still, it is not specific for knee OA [16]. This study showed that CTX‐II is expressed in female knee OA patients but not in males and could be gender specific. Compared to COMP, previous reviews concluded that CTX‐II was examined by studies with less potential bias. These reviews concluded that CTX‐II seemed to perform better as a diagnostic biomarker than COMP [10, 12, 34]. In the study included in this review, COMP was more reliable because it was not gender related [10].

The biomarkers of bone turnover, PINP, osteocalcin (bone formation) and CTX‐I (bone resorption), have also shown diagnostic value for early OA. Bone turnover is, besides cartilage degeneration, an important trademark of OA as a disease but not as reliable. The idea is that it is probably masked by the turnover of the whole skeletal system [34]. PIIANP and MMP‐3 have shown no correlation with early knee OA [10].

Urinary C2C is another marker of collagen type II degradation, meaning that C2C concentrations in the urine can directly reflect the decomposition of the articular cartilage. A study included in this review has shown that there is a significant difference in KL grade II, which is still considered early OA, compared to the control group. There was no difference in KL grade I compared to the control group [14]. C2C has the potential to detect OA changes, but only in the phase when there is radiographically visible damage. Also, another drawback is the nonspecificity of this biomarker joint‐wise, being also increased in hip OA [4].

A adropin blood level was found to be correlated with knee OA in a study included in this review. Unfortunately, this biomarker is not specific to knee OA because in previous studies, a lower blood adropin level was associated with obesity and ageing, which are both predisposing factors for knee OA [8].

There are other examples of nonspecificity of the biomarkers examined in the studies included, like calprotectin, an alarmin protein which is part of the general inflammatory response in the human body. In another study included in this review, calprotectin has shown higher expression in the early stages of OA, while in the later stages, its expression declined [30]. This is the desired pattern for the early knee OA diagnostic biomarker, as it is specific to the early stages of the disease. However, its disease specificity is not promising since it is elevated in immune‐inflammatory conditions (e.g., inflammatory bowel disease).

Another study that is included focused on angiogenic cytokines, which can also be considered inflammatory biomarkers [17]. This study showed levels of G‐CSF, HGF, angiopoietin‐2, follistatin and IL‐8 were significantly higher in early OA patients than in controls. One of the examined biomarkers in this study, follistatin, could be proven useful because its concentration was increased in early OA compared to healthy controls, but it decreased in advanced knee OA, meaning that its high expression can be specific for early knee OA. All of these angiogenic factors are not specific to knee OA since they are present in a plethora of other inflammatory conditions.

In the last two decades, it was realised that OA is not merely a ‘wear and tear’ disease of synovial joints and that chronic joint inflammation plays a crucial role in joint destruction [31]. Studies have shown that early knee OA expression of inflammatory factors is even greater than in late knee OA, caused by ongoing synovitis [3, 21]. Inflammatory markers seemed suitable to detect early knee OA because the inflammatory process precedes articular deterioration. Therefore, inflammatory markers should also precede cartilage degradation products, which are ‘downstream’ products of inflammation [27]. Unfortunately, no OA‐specific inflammatory biomarker was found.

If a putative biomarker is being tested as an indicator of disease, then it needs to be compared to an independent ‘gold standard’ measure of disease [6]. One of the greatest inconsistencies that is commonly met in assessing biomarker performance is that it is usually compared to relatively simple radiographic measurements [19]. The KL classification is not sensitive enough to early knee OA. This classification is based on a subjective grading, which makes it prone to observer bias. The objective drawback is that standard weight‐bearing radiography visualises bone and can only approximate cartilage thickness and does not evaluate synovium at all. Joint line narrowing can be a result of meniscal extrusion and not just cartilage deterioration [6, 18]. The further limitation of standard radiography is radiation exposure. Magnetic resonance and arthroscopy can provide more valuable and representative information, but the cost‐efficiency and invasiveness, respectively, of these procedures are objective obstacles. It is doubtful that magnetic resonance has any advantage in diagnosing OA compared to weight‐bearing radiographs. Bone marrow lesions that can be seen on MRI can detect early knee OA and be correlated with certain bone turnover biomarkers. In this systematic review, only X‐ray outcome measures were taken to obtain more homogenous results; arthroscopic, MRI and clinically diagnosed early knee OA were excluded. This is one of the main reasons for such few studies included taking only X‐ray classification due to heterogeneity in early OA definitions. With more studies with MRI classification, a more precise diagnosis of early OA could be achieved.

The most promising diagnostic biomarkers for OA are still in their explorative stage. The comparison of biochemical markers' performance is a complex task due to the uneven standard of measurements across markers and the heterogeneity of study designs. Another great obstacle is that most OA biomarkers are not specific to a certain joint, and OA can be a generalised disease [6]. Also, both cartilage and bone turnover biomarkers from osteoarthritic joints overlap with the physiologic turnover occurring in the whole organism. This turnover can also be increased in other arthritic diseases. Another difficulty when comparing biomarkers to radiological grade is that biomarkers have a phasic activity that is not always in line with a relatively linear radiologic progression of OA. Soluble biomarkers are more reflective of disease activity than current radiological disease status [13]. Imaging parameters lack a direct correlation with joint function and pain, which represent the crucial clinical importance for the individual patient with OA [28].

There is a clear rationale behind biochemical markers' role in early diagnosis of OA. Scientists are constantly striving to find the right biomarkers, but breakthroughs in the biochemical marker field are scarce, and no great breakthroughs have been made. Some authors are pessimistic about the future applicability of biochemical markers for OA diagnosis [6]. There are various factors that may contribute to this, including differing outcome definitions for incidence and progression, inadequate and inconsistent evidence for most available biochemical markers, lack of optimisation of data collection methods and a shortage of well‐designed large prospective studies that enable controlling for confounding factors [9].

Previous systematic reviews agree that neither of the commercially investigated biomarkers had diagnostic or prognostic accuracy needed for use in clinical practice [5, 9, 16, 27, 34]. Some of the previous systematic reviews were only about prognostic biomarkers or had different biomarker classifications than this review. Still, the main difference between this review from previous ones was that most of the previous ones did not differ early from an advanced stage of OA. The opinion of this study group is that advanced OA is very easily diagnosed clinically and radiographically, but there is a need to find a biomarker that can diagnose the early stage of the disease when structural changes could be prevented and the vicious circle of disease broken.

The present review is also in agreement with the idea that no currently known single biomarker is adequate for diagnosing early knee OA. There is evidence that a cluster of different biomarkers should be used in order to increase sensitivity and specificity, with at least one of them being from cartilage metabolism and one inflammatory [16, 22, 27]. This strategy could provide more consistent results than a traditional ‘one biomarker—one disease’ approach.

If a biomarker can reliably detect the early phase of the disease (subclinical), it could enable physicians and physiotherapists to take preventive measures and apply disease‐modifying treatments. Some authors have called this a ‘personalised prevention’ strategy [29]. Most patients do not seek any kind of medical consultation or treatment until symptoms become evident and unbearable. This reliable biomarker could also be used as an outcome measurement for future drug development, especially in the field of disease‐modifying agents. Since orthobiologic therapies have shown promising effects [15], a suitable biomarker which would detect patients with preclinical knee OA would enable their use to prevent the loss of joint function.

## CONCLUSION

The opinion of this study group is that there is no reliable biomarker available that could differentiate between early knee OA patients and healthy controls. There is a potential role for a cluster of biomarkers to be able to fulfil this role. Future studies should thus examine groups of biomarkers together, without focusing on just a single biomarker.

## AUTHOR CONTRIBUTIONS

Marko Ostojic wrote the first draft of the paper and interpreted the data. Joao Pedro Oliveira and David Kordic did the title and abstract screening and extracted the data. Robert Prill was responsible for and wrote the methods of study. Caroline Mouton did data curation. Caroline Mouton and Roland Becke did the revision of the first draft. All Authors revised the manuscript critically for important intellectual content.

## CONFLICT OF INTEREST STATEMENT

The authors declare no conflict of interest.

## ETHICS STATEMENT

The authors have nothing to report.

## Data Availability

Data are available on request from the corresponding author.
